# The detection of cardiovascular biomarkers in dermal interstitial fluid - a step to real-time monitoring

**DOI:** 10.3389/fcvm.2026.1724051

**Published:** 2026-07-13

**Authors:** Yousaf Bhatti, Alireza Yazdi, Daniel A. Jones, Didier Locca, Pankaj Vadgama, Adrian Mihai Ionescu, Anthony Mathur

**Affiliations:** 1 Barts Heart Centre, Barts Health NHS Trust, London, United Kingdom; 2 Centre for Cardiovascular Medicine and Devices, William Harvey Research Institute, Queen Mary University of London, London, United Kingdom; 3 NIHR Barts Biomedical Research Centre, Queen Mary University of London, Charterhouse Square, London, United Kingdom; 4 Institute of Electrical and Micro Engineering, Swiss Federal Institute of Technology-EPFL, Lausanne, Switzerland; 5 School of Engineering and Materials Science, Queen Mary University of London, London, United Kingdom; 6 École Polytechnique Fédérale de Lausanne (EPFL), Nanoelectronic devices laboratory, Lausanne, Switzerland

**Keywords:** biomarkers, digital algorithms, precision medicine, sensing, wearables

## Abstract

Cardiovascular biomarkers are central to the diagnosis and management of a range of acute and chronic disease states, yet current approaches remain dependent on episodic venous blood sampling. Dermal interstitial fluid (ISF), a dynamic and accessible biofluid, offers a promising alternative for continuous and minimally invasive biomarker monitoring. In this Review, we explore the emerging convergence of ISF biosensing technologies with cardiovascular diagnostics. We discuss the physiological basis of ISF sampling and highlight engineering advances in microneedle arrays, hydrogel implants and electrokinetic extraction methods. We examine state-of-the-art biosensing platforms—ranging from enzymatic electrochemical sensors to aptamer- and antibody-based systems—and evaluate their applicability to key cardiovascular biomarkers, including troponin, natriuretic peptides, CRP and microRNAs. Although early studies demonstrate proof-of-concept for ISF-based detection of several cardiac biomarkers, clinical translation remains limited. While still in proof of concept stage, continuous ISF monitoring could disrupt traditional models of care by enabling earlier detection, personalised risk stratification and remote disease management. However, it also presents challenges related to clinical workflow integration, data interpretation and regulatory oversight. Bridging these gaps will require multidisciplinary innovation across materials science, device engineering and clinical validation. By transitioning from episodic blood sampling to continuous dermal monitoring, this technology promises to redefine cardiovascular care; shifting the paradigm from reactive acute management to proactive, personalised, and predictive precision medicine.

## 1 Introduction

Cardiovascular disease (CVD) is the leading cause of mortality worldwide with an estimated 19.8 million deaths in 2022 alone (1). Reducing CVD mortality has been a key objective of healthcare organisations globally, with the World Health Organisation (WHO) targeting early disease detection as an area for improvement (2). To address this global burden, clinical focus has shifted towards improved diagnostic precision. Early detection enables the prevention and optimal management of CVD to reduce morbidity and mortality. Despite the utility of venous blood sampling, current diagnostic workflows face significant limitations, particularly in the ‘indeterminate’ patient group. In the context of acute coronary syndromes, a significant proportion (15%–40%) of patients present with troponin levels that are neither clearly normal nor diagnostic of infarction, necessitating prolonged hospital observation and repeat blood draws (3, 4). This diagnostic uncertainty places a substantial economic and logistical burden on healthcare systems (5, 6). Continuous monitoring of biomarker trends in dermal ISF is hypothesised to offer a potential solution to this ‘clinical grey zone,’ providing high-resolution temporal data that could differentiate acute pathological release from chronic background elevation earlier than episodic venous sampling.

Among the key strategies for early detection are techniques based on biomarker monitoring. Biomarkers are measurable indicators of biological processes, pathological states, or responses to interventions (7) and provide objective and quantifiable information about cardiovascular health. They can be molecules such as proteins, metabolites, or nucleic acids found in blood, interstitial fluid, saliva, or other biological fluids. In the context of health monitoring and diagnostics, biomarkers provide critical insights into the state and dynamics of a person's health. There are several well-established cardiac biomarkers that enable clinicians to detect and monitor disease. They are used in the acute (troponin, BNP), sub-acute (troponin, BNP, CRP, glucose,) and chronic (BNP, lipids, HbA1c, iron studies) setting. Monitoring of these biomarkers play a central role in international clinical guidelines (8). Biomarkers are typically detected from venous blood samples, requiring the associated healthcare resources (9, 10). However, biomarkers are detectable in a wide range of biofluids including dermal interstitial fluid (ISF). Comparative to venous sampling, skin is readily accessible, and sampling is minimally invasive for patients.

The use of continuous glucose monitoring via ISF in the management of diabetes has been an exemplar that has demonstrated validity with gold standard measurements and improved patient outcomes in clinical practice (11). Recent studies show that understanding the biochemical relationship between serum and dermal ISF is critical for advancing minimally invasive diagnostics and demonstrated that establish dermal ISF as a promising matrix for clinical application (12). There is now opportunity to apply these advantages to CVD detection and management.

To date, there are no clinical studies evaluating the use of continuous subcutaneous monitoring of cardiac biomarkers in human subjects. This novel sampling route represents a new area of research for CVD. There are many potential advantages over traditional blood-based biomarker measurement continuous sampling and detection will be explored in this review. Dermal ISF cardiac biomarker measurement offers the opportunity to improve diagnostic accuracy, widen diagnostic windows, elicit novel biological insights and generate larger data sets with greater temporal resolution for machine learning algorithms; though these benefits will need to be clinically validated.

In this review we interrogate the current state of research for sampling and detecting cardiac biomarkers in dermal ISF with the aim of exploring the next steps towards the development of continuous wearable sensors for clinical applications.

We review current sensor technology and the most viable sampling and measurement methods. Specific cardiac biomarker characteristics are summarised with a focus on the potential application of current sampling and monitoring technologies. Finally, we will review how continuous sensors might revolutionise cardiac care in the future.

### 1.1 Composition and role of dermal interstitial fluid

Interstitial fluid (ISF) is present in all extracellular environments in the human body. It has traditionally been characterised as a fluid reserve for blood volume functioning passively. However, there is an increasing body of evidence that indicates it is in fact a dynamic biofluid involved in the regulation of multisystem processes.

ISF is the largest volume fluid compartment and the most accessible biofluid in the human body (13). For the average 70 kg man, 60% of the total body weight is comprised of water, equalling 42 L; out of the extracellular fluid volume, approx. 75% or 10.5L of the volume is present in the interstitial space, and approx. 25% of that water is in the plasma, equivalent to 3.5L (14). ISF is found in visceral organs, the central nervous system and the lymphatic system. It is most abundant and accessible in subcutaneous tissues. As summarised in Table 1, when compared to other non-invasive biofluids such as saliva or urine, dermal ISF may offer the optimal balance of continuous wearable compatibility and biomarker concentration fidelity. Notably, while saliva and urine successfully harbour cardiovascular biomarkers like NT-proBNP and Troponin, their concentrations are significantly lower or heavily dependent on filtration rates, making them less suitable for the real-time, high-resolution monitoring required in acute cardiovascular care.

**Table 1:** A comparison of human biofluid sampling characteristics for key cardiovascular biomarkers.

Biofluid	CV biomarkers detected	Relative concentration (vs. venous blood)	Continuous wearable compatibility	Translational readiness
Venous Blood	Troponin, NPs, CRP, cMyBP-C, Lactate	Baseline reference concentration	Low (Requires invasive venepuncture; episodic)	Established Clinical Gold Standard
Dermal ISF (15–21)	Troponin I, Lactate, Lipids, microRNAs	Size-dependent: Small molecules (e.g., lactate) ≈ 100% of blood; Proteins (e.g., Troponin, CRP) are significantly attenuated. Larger proteins subject to lag times.	High (Microneedle arrays, transdermal optical)	Emerging (Human pilots for Lactate and Troponin)
Saliva (18, 22–27)	NT-proBNP, CRP, Troponin, microRNAs	Significantly lower (e.g., >1000-fold lower for NT-proBNP)	Moderate (Intraoral sensors emerging, but prone to fouling)	Research/Episodic screening
Urine (18, 28–30)	NT-proBNP, Troponin, microRNAs	Variable (Highly dependent on renal filtration rates)	Low (Not physically suitable for continuous real-time sensing)	Validated for episodic community screening
Sweat (20, 31)	Lactate, Sodium (20, 31)	Variable (Poor correlation with blood for large proteins)	High (Epidermal patches)	Validated for metabolites; unproven for CV proteins

**Figure 1:**
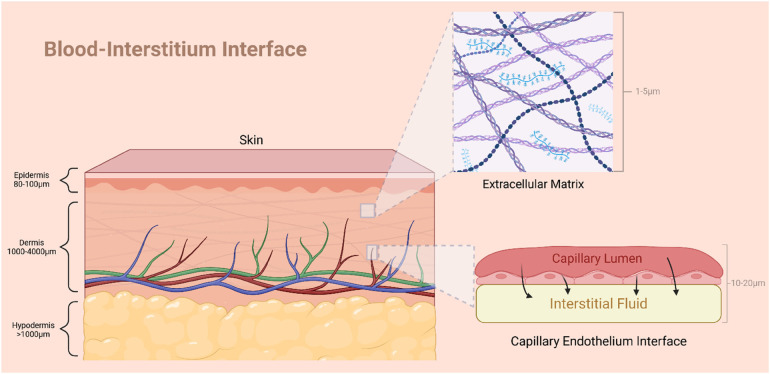
A schematic diagram of the blood-interstitium interface. Vascular and lymphatic systems interact in the dermis via interstitial fluid which runs through the mesh network of the extracellular matrix. The interface is regulated by physiological flow and permeability of the vascular endothelium. The schematic reflects the dermal layers with magnifications of the dermis detailing the gelatinous nature of the extracellular matrix (upper) and the capillary endothelium interface regulating dermal interstitial fluid and blood transport. Created in https://BioRender.com.

The dermis and hypodermis constitute the thickest layers of skin and sit beneath the avascular epidermis, as illustrated in Figure 1. The vascular and lymphatic systems sit within this layer interacting through ISF that permeates the gel like extracellular matrix (ECM) (see magnified boxes in Figure 1 (32). The ECM is made up of a 3D mesh of specialised cells, collagens, laminins and proteoglycans which can regulate ISF contents (33). ISF functions as a dynamic fluid medium through which nutrients, waste, immune mediators and hormones are transported through the interstitial ECM system. ISF content is dependent on whole body physiology and is replenished every ∼24–48hrs (32, 34). Importantly, ISF contains no coagulation factors. This makes *in-situ* continuous sampling and detection attractive – a key advantage over blood sampling.

The volume of ISF follows the Starling mechanism and is determined by the balance of transcapillary hydrostatic and osmotic pressure, and the permeability of capillary cells and lymphatic drainage (34). The transport of molecules into the ISF is determined by both diffusion and fluid flow (13, 35). Small to medium molecules up to ∼70 kDa readily transfer through paracellular routes. Thus, small molecule and ion concentrations are comparable in ISF and intravascular fluid. Several comparative metabolite and protein studies have demonstrated minimum constituent differences between ISF and vascular serum, albeit at lower relative concentrations (36–39). Therefore, many established and emerging cardiovascular biomarkers of small molecular size are likely to be present in ISF and are reviewed in detail later.

**Figure 2:**
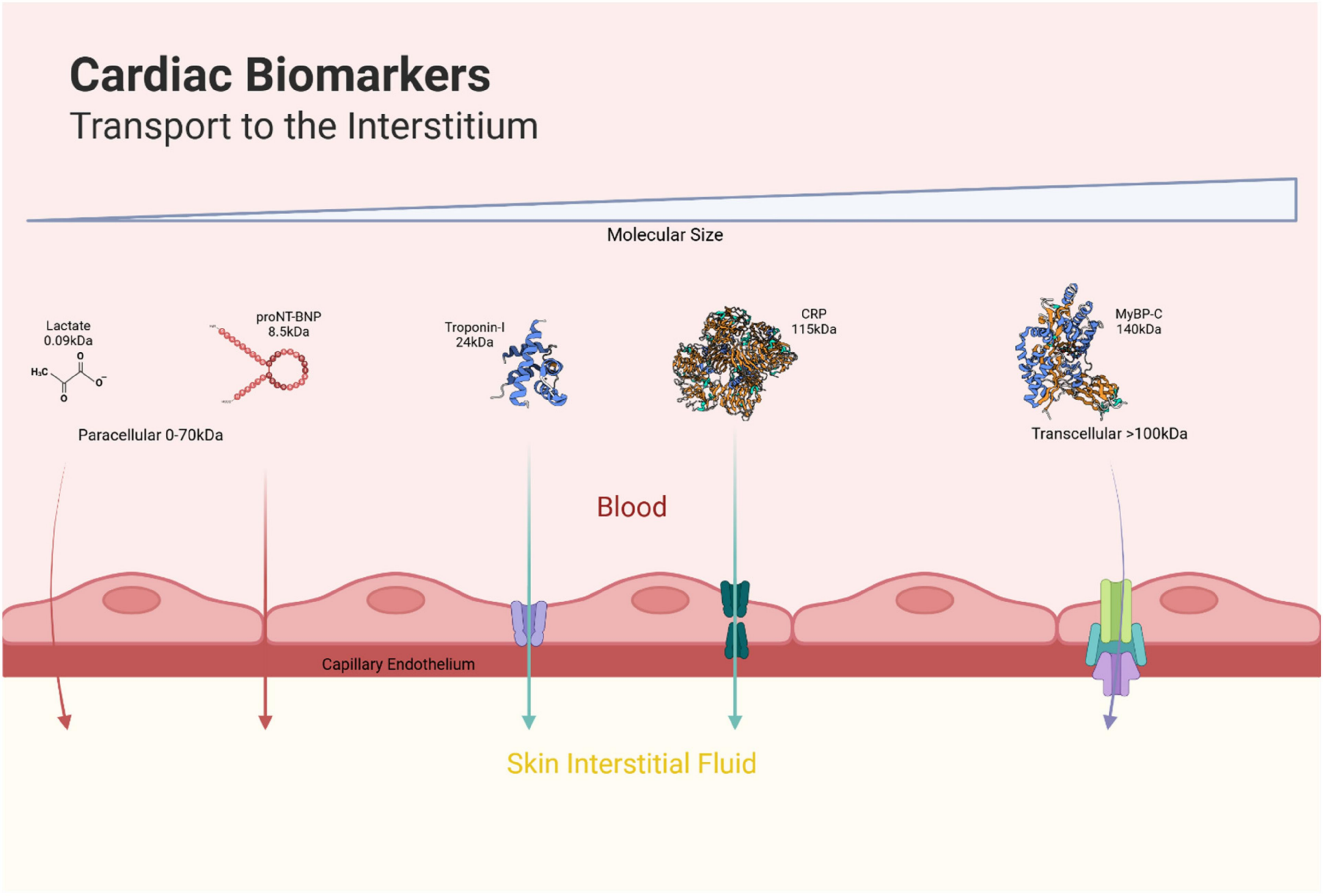
A schematic diagram summarising cardiac biomarker transport to the interstitium. The diagram shows the conceptual model of transport of proteins across the vascular endothelium to dermal interstitial fluid which is largely dependent on biomarker size characteristics. Transport is either paracellular or transcellular, with some biomarkers using both pathways. Small metabolites such as lactate (0.09 kDa) are able to diffuse freely across the membrane; medium sized molecules such as troponin (18-24 kDa) and C-reactive protein (CRP)(115 kDa) are able to transport across the membrane through paracellular or transcellular transporters driven by concentration gradients. Larger molecules such as Myosin-Binding Protein C (MyBP-C)(140 kDa) require more complex membrane transporters. proNT-BNP, N-terminal pro-B-type natriuretic peptide; kDA, kilodalton. Created in https://BioRender.com.

Larger molecules >100 kDa are dependent on transcellular processes but remain subject to gradients in concentration and fluid pressure. It is important to note that permeability of the ISF-blood barrier is not fixed and can be affected by inflammation, paracrine mediators and mechanical factors. Transport of these molecules into the ISF is governed by size-dependent paracellular or transcellular mechanisms, establishing ISF as a dynamic reservoir with a composition comparable to blood. The likely mechanisms of transport of key cardiovascular biomarkers is summarised in the conceptual model diagram (see Figure 2).

## 2 Methods for sampling dermal interstitial fluid

### 2.1 ISF extraction methods

**Figure 3:**
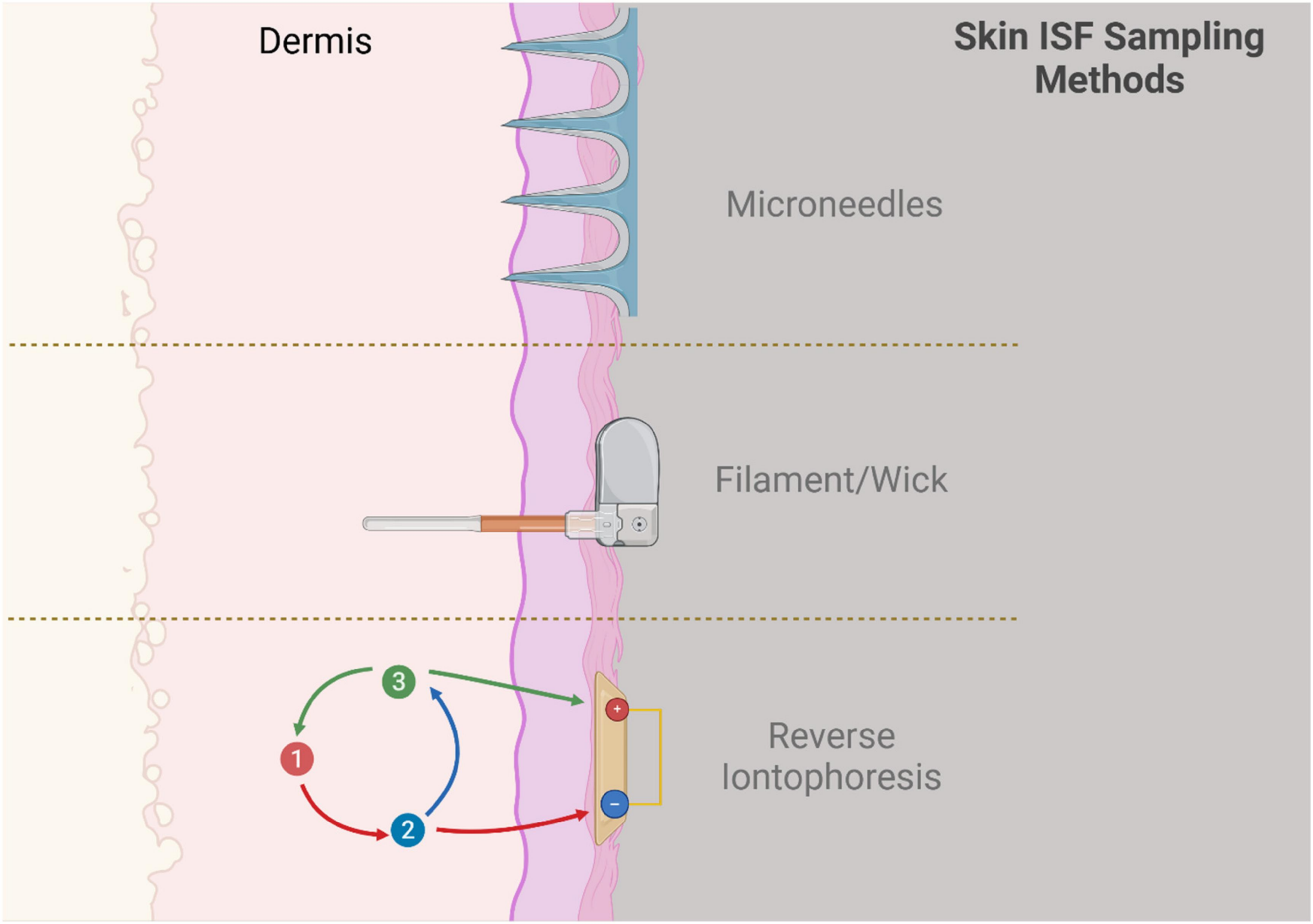
A schematic diagram of dermal interstitial fluid sampling methods. The diagram summarises 3 common methods of dermal interstitial fluid sampling. Hollow microneedle array sampling (top), filament/wick sampling (middle), reverse iontophoresis (bottom). ISF, interstitial fluid. Created in https://BioRender.com.

There are several methods of ISF extraction described in the literature and available commercially (see Figure 3). Advantages of dermal ISF sampling include minimal invasiveness, simple use, continuous sampling and coupling to microchips and sensors. Key challenges to extraction are low volume acquisition, target protein concentration, extracellular matrix effects and tissue disruption. ISF extraction has been reviewed in detail by others (40), and a relevant summary for this review is outlined below:

### 2.2 Microneedles

**Figure 4:**
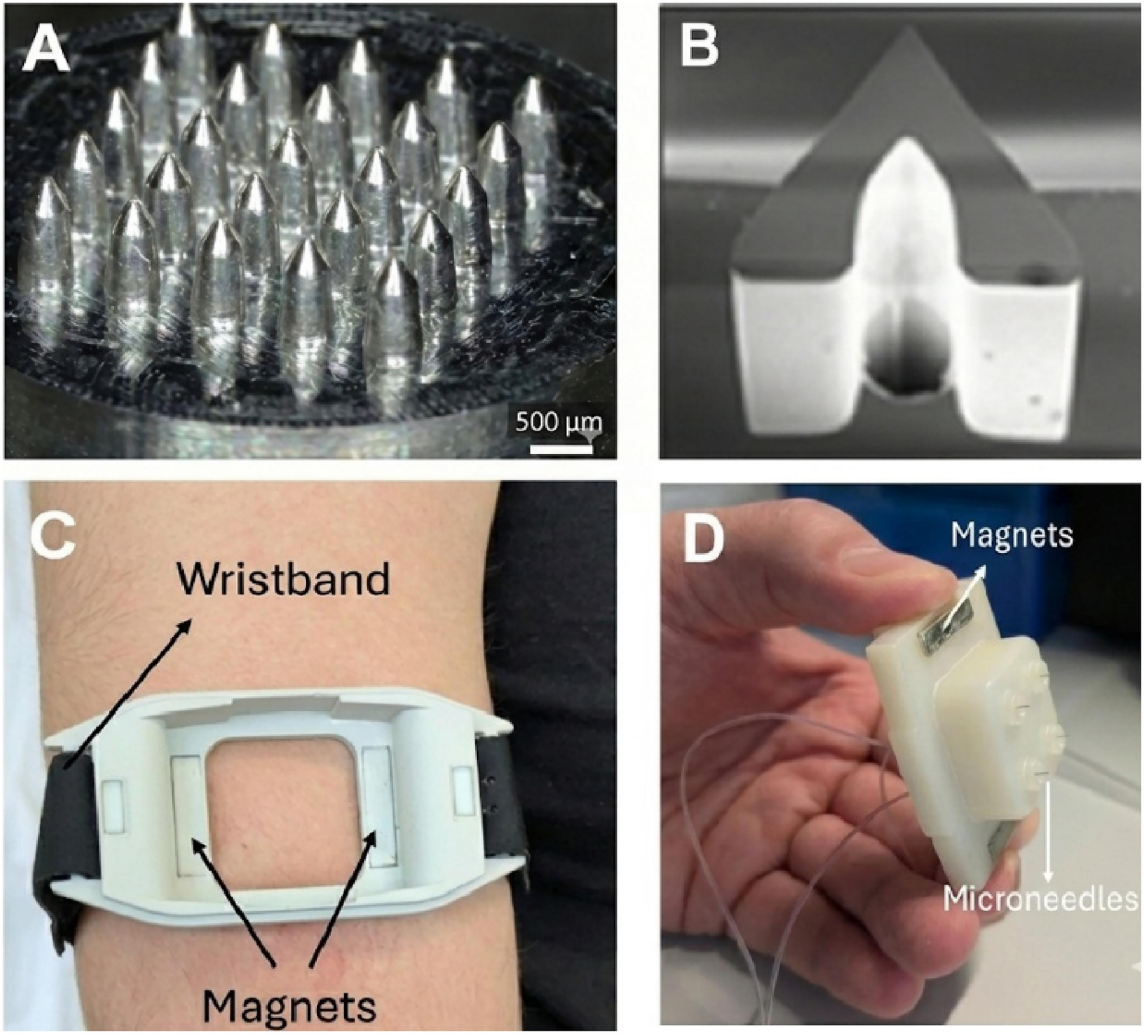
Photomicrographs and photographs showing technological applications of hollow microneedles for interstitial fluid extraction. **(A)** Self-extracting dextran-based hydrogel microneedle arrays microneedles. Reproduced from “Digital images for (A)” by Bastien Darmau, Marta Sacchi, Isabelle Texier and Andrew J. Gross, licensed under CC BY 4.0. **(B)** Ascilion's silicon microneedles. Adapted from “Photos of manufactured sharp hollow microneedles in monocrystalline silicon. **(a)** Scanning electron microscope picture of an individual microneedle” by Markus Renlund, Laurenz Kopp Fernandes, Pelle Rangsten, Mikael Hillmering, Sara Mosel, Ziad Issa, Volkmar Falk, Alexander Meyer and Felix Schoenrath, licensed under CC BY. **(C)** Xsensio's magnetic wristband cassette holder. Reproduced from “Xsensio's dermal interstitial fluid (dISF) collector. (a) Base component of the dISF collector, serving as a support structure with a wristband on both sides to apply pressure and embedded magnets to attach to the collection component” by Yann Sprunger, Johan Longo, Ali Saeidi, and Adrian M. Ionescu, licensed under CC BY. **(D)** Xsensio's metal microneedle cassette. Reproduced from “Xsensio's dermal interstitial fluid (dISF) collector. (b) Collection component featuring BDTM 31G microneedles connected to TygonTM tubes for dISF extraction“ by Yann Sprunger, Johan Longo, Ali Saeidi, and Adrian M. Ionescu, licensed under CC BY.

This method is a minimally invasive technique that utilises arrays of micron-scale projections, typically ranging from 150 to 1500 µm in length with tip diameters <20 µm, to penetrate the stratum corneum and access the dermal ISF without stimulating deep nociceptors (see Figure 4) (12, 38, 41–43). Microneedles for ISF (44) should fulfil certain well specified features when applied against the skin, such as penetration depth, insertion and fracture force, mainly related to their material mechanical properties, while remaining biocompatible; they are typically made of metal, silicon or polymers, forming body interfaces of adhesive patches that enable continuous sampling of dermal ISF. Polymers are claimed to provide better solutions for safer and longer-term biomarker monitoring and, in some cases, they come with transient self-dissolving properties. The application is typically painless provided certain conditions for the diameter of the individual needle are respected (45). It is worth noting that there are multiple types of microneedle architectures and strategies to access the ISF: (i) hollow microneedles (15), essentially aiming to extract small volumes of ISF for analysis with sensors operating outside the body, (ii) porous microneedles utilizing capillary-driven channels to extract ISF (46), and, (iii) solid-state microneedles with embedded sensory layers, enabling measurements in ISF directly under the skin, removing the need of ISF extraction (47). Despite many recent advances, microneedle patch technology is mostly used for research purposes. Further development of manufacturing processes and biosensor coupling is needed to reach clinical and commercial applications.

### 2.3 Electroosmotic/reverse iontophoresis

Reverse iontophoresis is a non-invasive collection method that draws ISF to a collection system by applying a driving current across two nodes (48). This method has been utilised in past commercially available continuous glucose monitors (49). The method has also been utilised to collect troponin and microRNA samples (16). A key limitation of this technique is the exclusion of larger biomarkers and potential interference from sweat, and the effects of, skin temperature and hydration status.

### 2.4 Filament/wick/needle

This group of methods work by collecting ISF through a hollow tube that sits within the dermis. Depending on the method, ISF is drawn through a tube by capillary action, skin suction or external pressure. This method allows for *in-situ* detection and has been shown to work in humans for several days without need for renewal. This method is currently used in a commercially available continuous glucose sensor (50–52).

### 2.5 Emerging extraction techniques

There are several other methods of ISF sampling described in the literature that require further development to be adopted into wearable or clinically used devices. Suction blister extraction, for example, uses negative pressure and heat to separate the epidermis from the dermis, creating a blister filled with ISF. While effective for obtaining larger volumes for proteomic analysis (36, 37), it is generally too invasive and time-consuming for continuous wearable applications. Other emerging techniques include microdialysis and hollow micro-capillary loops, which allow for continuous perfusion and sampling but currently require external pump systems that may limit ambulatory use (53–55).

## 3 Biorecognition-layer-driven approaches to interstitial fluid sensing

Detection of biomarkers in the dermal ISF requires modification of current technologies to meet the requirements of repeated *in-situ* testing. ISF offers the advantages of being an easily accessible and non-coagulable biofluid; this eliminates most pre-processing required for laboratory blood tests. However, detection methods must overcome low concentration levels, extracellular matrix effects and repeat detection stability.

Both electrical and optical modalities have demonstrated significant potential for the non-invasive monitoring of biomarkers in dermal interstitial fluid (ISF) within wearable continuous monitoring platforms. Optical approaches, benefiting from high intrinsic sensitivity, can in principle achieve lower detection limits. However, their integration into miniaturized, low-power wearable form factors remains challenging due to constraints in optical component miniaturization, power consumption, and signal stability under dynamic, real-world conditions. Consequently, optical methods are currently more suited to point-of-care (PoC) applications, where compactness and continuous operation are less critical.

In contrast, electrical detection technologies have matured to a stage where they can be readily implemented in scalable, low-power wearable systems taking advantage of silicon CMOS technologies. These methods rely on monitoring electrical signals—either direct current (DC) or alternating current (AC) responses—in passive elements or active devices such as field-effect transistors with integrated functional layers. In all cases, specificity arises from a functional biorecognition layer that transduces the binding of the target analyte into a quantifiable electrical signal proportional to its concentration. Electrical ISF sensors utilise a diverse range of transduction mechanisms to convert biorecognition events into quantifiable signals. While early wearable platforms predominantly utilised amperometric enzymatic detection, recent advances have expanded the sensor landscape significantly. Current architectures include, but are not limited to: (a) enzyme-based sensors, which exploit catalytic turnover for signal amplification; (b) ion-selective electrodes (ISEs), which respond to changes in ionic activity; (c) affinity-based sensors utilizing (c) aptamers or (d) antibodies for detection. Other technologies such as field-effect transistors (FETs), which offer label-free, high-sensitivity detection by modulating channel conductance upon analyte binding have been described (56). Furthermore, emerging platforms are increasingly integrating impedance-based and CRISPR-mediated sensing modalities to enhance specificity for complex nucleic acid targets in ISF (57).

### 3.1 Enzyme linked sensors

Most of the CE-marked/FDA approved or commercially available wearable ISF monitors use enzyme linked sensors. These sensors use immobilised enzymes that catalyse reactions with the target analyte. The enzyme can be immobilised to a molecular ‘wire’ or redox polymer which acts to facilitate electron transfer from the enzyme active site to the electrode generating a signal proportional to analyte concentration (50, 58). This method of detection is the predominant technology found in commercially available dermal sensors that have been clinically validated (glucose) or are under clinical evaluation (lactate) (58–66). This method relies on analyte-enzyme specificity which is not available for larger molecules such as troponin or BNP. This may be overcome by antibody-enzyme conjugation, similar in principle to widely used enzyme linked immunosorbent assay (ELISA) based tests. However, these types of sensors have been mainly demonstrated for biomarkers having high concentration levels (metabolites).

### 3.2 Ion-sensitive electrodes (ISEs) and membranes (ISMs)

ISEs can measure pH and specific ion concentrations in ISF. They work using an ion selective membrane that generates a potential difference detected by an electrode (67). This sensor type is capable of detecting physiologically important ion concentrations continuously (22, 31, 68, 69). ISE use is limited to ion concentrations in the dermal ISF, which is relevant in heart failure patients. Peripheral oedema is a relatively late clinical sign of heart failure. It is caused by an expansion and change in ionic composition of ISF (70). ISE based sensors may therefore offer a more sensitive and early detection of haemodynamic changes driving these factors. It is also possible to couple thermistors and monometers that can measure dermal temperatures and pressures; these may offer further insight into heart failure related oedema mechanisms. The ISMs with specific ionophores are particularly suited for multi-modal detection of electrolytes in almost any types of biofluids, including ISF, being particularly scalable in terms of size and low volumes to be analysed. For instance, the multiplexed detection of Na^+^, K^+^, Ca^2^^+^ and pH has been demonstrated by Zhao et al. in a fully integrated CMOS chip with back-end-of-line integration of ISMs on metal electrodes achieving excellent sensitivity and selectivity together with ultra-low power consumption. Similar works combining metabolite and ion detection demonstrated the superiority of electrical methods combining ISE/ISM and enzymatic sensors. Translating this to a wearable format, Parrilla et al. developed a wearable, all-solid-state potentiometric microneedle patch specifically for intradermal potassium detection, demonstrating that ISEs can be successfully miniaturised for direct ISF electrolyte monitoring without fluid extraction (47).

### 3.3 Aptasensors

The aptamer-based sensor (Aptasensor) is an emerging technology that has several advantages for application in wearable monitors. Aptasensors utilise short oligonucleotide structures (aptamers) that have high specificity and affinity for a range of potential biomarkers including small molecules, ions, proteins and pathogens (71–74). These versatile aptamers are synthetically manufactured and can bind to any desired target. Analyte binding generates a conformation change in the aptamer that can be measured electrically, optically or by mass, compatible with existing detection methods. A typical example of electrical detection based on the functionalization of graphene electrodes with aptamers and detection with a subsequent field effect transistor has been reported for the detection of a small molecule like cortisol (75).

A key advantage of this technology is that it relies on the intrinsic aptamer-target binding properties that can enable label-free detection (76). This approach design avoids the need for additional tagging to enzymes, fluorescent dyes or nanoparticles for signal generation. In addition, nucleic acids are less immunogenic and have improved stability over antibodies.

These properties make aptasensors ideal for use in wearable sensors that require repeat analyte interactions over longer periods of time. However a key challenge is the potentially reduced analyte-aptamer interaction in the complex ECM which may interfere with binding. A solution to non-specific response is the use of a dual-sensor aptasensor architecture that subtracts the signal from a reference sensor operating on the same substrate and functionalized with a mixed aptamer, which allows for non-specific binding and drift corrections (75).

In the cardiovascular domain, aptamer technology has successfully been used to detect troponin, natriuretic peptides, myoglobin and CK (77–87). However these methods remain experimental and have mostly been applied to serum samples ex-vivo. The application and performance of these cardiovascular biomarker DNA aptamers is relatively untested *in-vivo*.

### 3.4 Antibody sensors

The antibody-based sensor (immunosensor) is a mature and widely used technology that leverages the high specificity and affinity of antibodies for their target antigens. Antibodies are Y-shaped proteins produced by the immune system that can bind a wide range of analytes, including proteins, peptides, small molecules, and pathogens, with remarkable selectivity. In biosensing applications, antibodies (or engineered antibody fragments, sometimes called nanobodies) are immobilized on a transducer surface, and analyte binding is transduced into an electrical, optical, or mass change signal, compatible with various detection platforms.

A major advantage of antibody-based sensors is their broad availability and well-established biofunctionalization chemistry, enabling their integration into a wide variety of substrates and device architectures. Antibody fragments, in particular, can enhance sensor performance by reducing the Nerst limit and improving binding kinetics. This makes immunosensors well-suited for point-of-care diagnostics and potentially for wearable continuous monitoring, provided that surface regeneration strategies are developed to allow repeated analyte binding cycles. A representative example of electrical immunosensing is the label-free detection of C-reactive protein (CRP) using a double-gate silicon nanowire field-effect transistor (SiNW FET) sensor array functionalized with antibody fragments (88). In this work, the authors exploited a pseudo-super-Nernstian back-gate operation mode to achieve highly sensitive detection of CRP over clinically relevant concentration ranges without the need for labels or secondary amplification steps.

However, compared to aptamers, antibodies are more susceptible to denaturation under varying temperature, pH, or mechanical stress, and they require careful storage conditions to maintain functionality. In complex biofluids such as interstitial fluid, non-specific adsorption can affect accuracy, therefore, approaches such as subtracted reference sensing channels or antifouling surface coatings are commonly implemented.

## 4 Current commercial biosensing platforms

While the continuous monitoring of cardiac biomarkers represents a novel frontier, the fundamental technology for harvesting and sensing dermal ISF is already a mature clinical reality in metabolic medicine. The widespread adoption of Continuous Glucose Monitoring (CGM) has validated the ‘lab-on-skin’ concept, proving that ISF biomarkers can be reliably tracked over weeks to months with high clinical accuracy (31, 59–63, 89, 90).

As detailed in Table 2, the current commercial landscape is dominated by amperometric enzymatic sensors. Devices such as the Abbott FreeStyle Libre, Dexcom G-series, and Medtronic Guardian utilise a transcutaneous filament functionalised with oxidases (e.g., glucose oxidase) to generate an electrical current proportional to analyte concentration. More recently, fully implantable platforms like the Senseonics Eversense and Profusa Lumee have introduced optical detection methods utilising fluorescence and phosphorescence quenching, respectively—to extend sensor longevity up to 180 days.

**Table 2:** Commercially available dermal interstitial fluid devices with continuous monitoring.

Biomarker	Device/platform	Sampling interface	Detection method	Development status	Key references
Glucose	Abbott FreeStyle Libre 2/3	Subcutaneous Filament	Wired Enzyme Amperometry (Osmium mediator)	FDA/CE Approved	(50, 63)
	Dexcom G6/G7	Subcutaneous Wire	Enzymatic Amperometric (Glucose Oxidase)	FDA/CE Approved	(61, 90)
	Eversense E3 (Senseonics)	Subcutaneous Implant	Fluorescence (Boronic Acid Quenching)	FDA/CE Approved	(62, 89)
	Medtronic Guardian Connect	Subcutaneous Implanted electrode	Enzymatic Electrochemical	FDA/CE Approved	(60)
Tissue Oxygen	Profusa Lumee Oxygen	Hydrogel Implant	Phosphorescence Quenching	CE Marked	(91–93)
Ketones	Sibionics KS3	Transcutaneous enzymatic wire	Enzymatic amperometric (beta-hydroxybutyrate)	CE Marked (Consumer/Wellness)	(94, 95)

Critically, the commercial expansion of these platforms from glucose to other analytes is already underway. Regulatory bodies have recently granted CE marks for continuous ketone monitoring (e.g., Sibionics KS3) (94, 95) and tissue oxygen sensing (91–93), demonstrating the versatility of ISF as a diagnostic matrix. These FDA-approved and CE-marked devices serve as the foundational hardware upon which future cardiac-specific sensors (discussed in Section 5) will likely be modelled.

## 5 Cardiovascular biomarkers of interest

As discussed earlier, the transition of analytes from the bloodstream into the dermal ISF is mediated by the vascular endothelium and the extracellular matrix (ECM). The molecular weight and structure of each cardiovascular biomarker dictate its specific transport mechanism and, consequently, its likelihood of detection. As illustrated in Figure 2, smaller molecules largely permeate via passive paracellular diffusion, whereas larger protein complexes may rely on transcellular pathways. Understanding these transport kinetics is critical for interpreting the concentration gradients and time-lags inherent to ISF-based sensing. Here we review the literature relevant to how cardiac biomarkers might be detected in ISF and how continuous monitoring impacts clinical care.

### 5.1 Troponin

#### 5.1.1 Troponin structure and function

Troponin I (TnI) and Troponin T (TnT) are structural proteins of the cardiac sarcomere (24 kDa and 37 kDa, respectively) released into circulation following cardiomyocyte necrosis. Their relatively small molecular weight facilitates paracellular diffusion across the endothelial barrier, making them viable candidates for detection in the interstitial compartment (96, 97).

#### 5.1.2 Troponin in clinical practice

The detection of myocardial specific troponin subunits isoforms in blood serum underpins accurate diagnosis of acute myocardial infarction (AMI) (8). Since the development of the first troponin specific immunoassays nearly 50 years ago (98), much progress has been made to improve immunoassay sensitivity down to picogram per millilitre concentrations (99).

The development of high sensitivity troponin assays has enabled the rapid diagnosis of AMI. A disadvantage of high sensitivity assays is the detection of troponin levels that are of indeterminate clinical significance. AMI diagnostic algorithms make use of serial troponin testing to address this issue (8). There is emerging work looking at the clinical significance of troponin concentration trends in cardiac patients to further stratify risk (100–103). Repeated invasive troponin spot sampling is difficult, resource intensive and undesirable to patients. If successfully translated to clinical practice, continuous monitoring of troponin may therefore offer novel insight into personalised patient risk in the AMI group as well as wider cardiac diseases.

#### 5.1.3 Dermal interstitial fluid troponin

Troponin subunit molecular size ranges from 18 to 37 kDa, which is small enough to pass through the capillary membrane to ISF by passive paracellular route. Protein studies of ISF in tumour cell lines have identified many proteins of similar molecular size present in detectable quantities including tropomyosin *α* subunits (39). In a study looking at a novel electroosmotic method, TnI was detectable in the ISF of AMI rat models at pg/ml concentrations peaking 16 h after infarction (16). Interestingly the same study showed ISF concentrations of micro-RNAs peak much earlier which will be discussed later. A recent study has also shown detectable troponin in dermal ISF using a microneedle capacitive sensor. The platform utilised a 5 × 5 conical microneedle array functionalized with anti-cTnI antibodies to achieve label-free detection of cardiac troponin I directly in the interstitial compartment. The system demonstrated a dynamic range of 10 pg/mL to 10 ng/mL with a limit of detection (LOD) of 3.27 pg/mL and a rapid response time of under 15 min. Crucially, the study successfully validated the sensor's performance through spike-and-recovery tests in human serum and real-time monitoring in animal models, showing a high correlation with systemic blood levels (17). Troponin complex proteins are notably detectable in saliva, urine and amniotic fluid suggesting that the troponin subunits freely pass membrane barriers in the salivary glands, glomerulus and placenta (23, 28, 29). There is even evidence that troponin subunits can pass the highly regulated blood brain barrier (104). A US group has recently demonstrated the clinical feasibility of a wrist-worn transdermal optical sensor in identifying elevated troponin-I levels in patients with acute myocardial infarction, achieving an area under the curve (AUC) of 0.90 in validation cohorts (105). This proof-of-concept study supports the hypothesis that troponin permeates the dermal interstitium in detectable quantities during acute ischemic events.

To date, there are no dedicated studies to determine human concentrations of troponin subunits in dermal ISF by direct sampling. This remains an area for further study – of particular interest will be the trend of troponin over time in AMI patients and the temporal relationship with clinical events and symptoms. The physiology of ISF dictates that protein concentrations are likely to peak later than blood serum concentrations. However, this potential disadvantage may be overcome by high sensitivity detection methods already well developed for troponin. Additionally, continuous monitoring may offer novel trend insights over spot testing, which is discussed later.

### 5.2 B-type natriuretic peptide (BNP) and NT-proBNP

#### 5.2.1 Structure and function of natriuretic peptides

B-type Natriuretic Peptide (BNP, 3.5 kDa) and its N-terminal prohormone (NT-proBNP, 8.5 kDa) are small peptide hormones secreted in response to ventricular stretch (106, 107). Their low molecular mass allows for rapid equilibration between the vascular and interstitial compartments, theoretically minimising the ‘lag time’ to detection in the ISF often observed with larger protein biomarkers (97–99). To evaluate the feasibility of continuous cardiovascular monitoring, biomarkers must be prioritised based on their transport kinetics and existing validation data. Table 3 summarises these characteristics, highlighting the relationship between molecular weight and the primary mechanism of translocation into the dermal ISF. While small molecules like lactate and peptides like BNP exhibit favorable diffusion profiles, larger structural proteins such as cMyBP-C present significant transcellular transport challenges that may necessitate more sensitive sensing modalities.

**Table 3:** Translational readiness and transport characteristics of cardiovascular biomarkers in dermal ISF.

Biomarker	Molecular weight (kDa)	Primary transport mechanism	Current ISF evidence level	Priority application
Lactate	0.09	Passive Diffusion	Validated (Human research/Pilot) (20, 64–66)	Acute Shock; Ischaemia monitoring
BNP	3.5	Paracellular Diffusion	Research (Human Urine/Saliva proxy) (24, 30, 36, 108, 109)	Heart Failure Monitoring/Treatment Titration
NT-proBNP	8.5	Paracellular Diffusion	Research (Human Urine/Saliva proxy) (24, 30, 36, 108, 109)	Heart Failure Monitoring/Treatment Titration
microRNAs	∼7–8	Paracellular (Vesicular/Protein-bound)	Research (Animal models, Human proxy fluids) (16, 18)	Novel Ischaemia Detection
Troponin I	24	Paracellular	Human (Transdermal optical detection, proxy fluids)/Animal models (16, 17, 28, 29, 39, 104, 105)	Acute coronary syndromes
Troponin T	37	Paracellular	Human (Transdermal optical detection, proxy fluids)/Animal models (16, 17, 28, 29, 39, 104, 105)	Acute coronary syndromes
CRP	115	Transcellular/Transcytosis	Research (Human proxy fluids) (25, 26, 110–118)	Plaque stability; Chronic inflammation
cMyBP-C	140	Transcellular/Transcytosis	Theoretical	Early AMI detection (<3 h)

#### 5.2.2 The use of natriuretic peptides in clinical practice

The release of NPs by ventricular myocytes is in response to myocardial stretch - triggering a neurohumoral response to maintain cardiac output. This process is central to the pathophysiology of heart failure. BNP/NT-proBNP are a reliable diagnostic marker of heart failure with >90% sensitivity, but decreasing specificity with age (119, 120). NPs are cited in both the European Society of Cardiology and American College of Cardiology/American Heart Association practice guidelines for diagnosis. Notably the European guidance utilise NPs for exclusion, whilst American guidelines advocate NPs as part of inclusion criteria – reflecting the differing interpretations of specificity (121, 122).

The use of NPs expand to monitoring of cardiac remodelling, disease progression, response to treatment, prognostication, risk stratification and mortality (123–128). Some groups advocate serial testing of NPs in HF, although this remains an area under review. Nonetheless, current North American guidelines support the use of NP testing at the start and end of hospital admission as a minimum (122). The evidence therefore suggests that the use of serial NP testing in heart failure enhances diagnosis and management; thus continuous monitoring may lead to further improvements in these areas.

#### 5.2.3 Natriuretic peptides in biofluids

NPs are small peptides that are readily detectable in blood serum with several commercially available assays able to detect in the pg/ml range. NT-proBNP is a linear stable peptide with a longer half-life than BNP making it an ideal biomarker for detection in extravascular fluids (129). BNP and NT-proBNP are small peptides at 3.5 kDa and 8.5 kDa respectively meaning they can readily pass through the capillary membrane-dermal ISF barrier via the paracellular route. Their small size is likely to reduce the ISF matrix effects making detection in this fluid more viable. It is also important to note that extravascular volume (and therefore dermal interstitial fluid volume) is typically increased in heart failure pathophysiology leading to clinical oedema. This will increase the volume of ISF available for sample but the effect on peptide concentration is unknown.

To date there are no dedicated publications looking at the concentration or detection of NPs in dermal interstitial fluid specifically. However, numerous experiments characterising the proteomic contents of dermal ISF have found many proteins of similar molecular size and structure including proteins such as angiotensinogen relevant to heart failure (36). The absence of NPs from these studies, likely reflects the lack of cardiac disease in the population samples rather than absence of NPs in ISF specifically.

Nonetheless, there is good evidence that NPs are found in other extravascular fluid filtrates. Bellagambi and colleagues performed NT-proBNP analysis in saliva samples of acute heart failure patients (24). They showed a comparable relative decrease in saliva vs. blood NT-proBNP at discharge, reflecting successful treatment. Although the absolute concentration of NT-proBNP was 10^3^ lower in saliva, the trend of change was maintained; suggesting continuous monitoring of extravascular fluid yields results reflective of blood serum concentration changes.

Numerous studies have shown that NPs can be reliably detected in urine and correlates temporally with blood serum concentration trends (30, 108, 130). Non invasive detection has been adopted to improve community screening highlighting the wider applicability of the non-invasive biomarker approach (109).

### 5.3 C-reactive protein (CRP)

#### 5.3.1 Structure and function of CRP

C-reactive protein (CRP) is a highly conserved pentameric protein of the Pentraxin group of proteins. Human CRP is formed from 5 identical ∼23 kDa subunits forming a horseshoe structure of 115 kDa (131, 132). CRP is an acute phase protein produced predominantly by hepatocytes in response to interleukin activation and acute inflammation. Its function as an immune mediator and biomarker of inflammation is well characterised (110). The concentration of CRP is closely correlated to inflammation with acute rises seen in infection, malignancy and myocardial infarction.

#### 5.3.2 The use of CRP in clinical practice

CRP is a marker and mediator of myocardial infarction through inflammatory effects within the atherosclerotic coronary plaque. Temporal variability and lower sensitivity/specificity limit its use (on its own) as a diagnostic marker of acute myocardial infarction (AMI) (126, 133–135).

However, the development of high sensitivity CRP assays has enabled further understanding of chronic inflammation states relevant to atherosclerosis and plaque development. Thus, there is increasing evidence that CRP interpretation can aid cardiovascular disease prognostication with CRP correlating to mortality, infarction size and risk of heart failure (136–139).

These observations have led to clinical trials testing CRP guided treatment strategies. The JUPITER trial treated patients with elevated hs-CRP but normal LDL using rosuvastatin – demonstrating reduced major cardiovascular events (140). The CANTOS trial demonstrated reduced cardiovascular events in patients treated with the monoclonal antibody targeting interleukin-1β; the trial recruited previous AMI patients with raised CRP - supporting the inflammatory plaque hypothesis (141). Colchicine had been shown to reduce events in AMI patients, but this was not replicated in other trials (142, 143). There remains a lack of consensus on how to use CRP in clinical management and further study is required. Nonetheless continuous CRP monitoring (perhaps in combination with other analytes) offers the potential to monitor real time clinical risk and therapeutic response in cardiac patients.

#### 5.3.3 CRP in biofluids

Transport to ISF is dependent on both transcellular and paracellular diffusion making detection more difficult. However, the ISF membrane barrier becomes more permeable in inflammatory conditions which may facilitate higher ISF concentrations.

There is evidence that CRP is detectable in a range of biofluids supporting the hypothesis that CRP crosses the ISF blood barrier. CRP has been detected in pericardial fluid and correlates to myocardial infarction (111, 112). CRP is detectable in saliva and has been used as a non invasive test to screen for cardiovascular disease, periodontal disease and rheumatoid arthritis (25, 26). Several studies have looked at the detection of CRP in drain fluid - CRP is detectable in abdominal ISF and correlates to risk of anastomotic leaks after bowel resection (113). Chen and colleagues have shown CRP detectable in pleural ISF allowing the identification of empyema from parapneumonic effusions (144). There is further evidence that CRP is detectable in synovial fluid, amniotic fluid and highly regulated cerebrospinal fluid (114–118).

Although these studies show CRP is present in extravascular biofluids - there are no dedicated studies looking at the detection of CRP in dermal ISF. Furthermore, the detection of CRP in these fluids may reflect localised CRP mediated inflammation rather than systemic CRP concentration. Continuous ISF CRP detection may therefore have greater utility in non-cardiac conditions relating to infection, inflammation and malignancy.

## 6 Emerging biomarkers

There are many cardiovascular biomarkers in the research domain that are not in clinical use, but relevant to this review given their future potential. These biomarkers offer the potential of further diagnostic and pathophysiological understanding of cardiac disease; especially where there is uncertainty about optimal treatment such as – percutaneous coronary intervention (PCI) in non-ST elevation MI, PCI in chronic stable angina, management of chronic total occlusions, drug therapy for heart failure with preserved ejection fraction, asymptomatic severe aortic stenosis.

### 6.1 microRNAs

MicroRNAs (miRs) are short single stranded non-coding RNA molecules ∼22 nucleotides in length (145). They function as post-translational modulators of gene expression by partial or complete complementarity with target messenger RNAs (mRNAs) (146). There are over 2,500 human miRs catalogued with several hundred of interest as both biomarkers of cardiac disease and therapeutic targets (147). Study of miR expression in cardiac disease states have revealed novel mechanistic insights (148, 149). Of note miR-21 expression has been shown to mediate a number of cellular processes in ischaemic heart disease, cardiac hypertrophy and heart failure (150).

A linear miR of 22 nucleotides has an average molecular weight of ∼7–8 kDa. However, to prevent degradation miR are encapsulated within extracellular vesicles or bound to RNA-binding proteins in biofluids (145). This makes diffusion and transport to ISF dependant on cellular mechanisms. As discussed earlier, miR-499-5p is detectable in the dermal ISF, saliva and urine of rats (16). There is evidence that miR can be found in nearly all human biofluids including, amniotic/pleural/peritoneal/cerebrospinal fluid, tears and breast milk (18). Interestingly there is a largely equal distribution and quantity of miRs across these biofluids, that may reflect systemic concentrations of miR. Current detection methods rely on purification and amplification steps. A challenge of dermal ISF detection will be lower concentration and the lack of *in-situ* detection and quantification technology. Further elucidation of the relationship between cardio-specific miRs and disease is required as well study on dermal ISF miR quantities in cardiac patients.

### 6.2 cMyBP-C

Cardiac Myosin-Binding Protein C (cMyBP-C) is cardiac muscle protein that regulates interaction between myosin and actin to modulate muscle contraction force and velocity (151). It has a similar sensitivity and specificity for AMI diagnosis to troponin with better performance in early presenters <3 h of chest pain (152). cMyBP-C is a relatively large protein (140 kDa) with complex structure of 11 domains. Blood concentration after AMI ranges from 120 to 900 ng/L; intact dermal ISF concentrations are likely to be limited by size dependant transcellular diffusion. To date, there are no studies looking at non vascular biofluid detection of cMyBP-C and this is an area for future study.

### 6.3 Lactate

Lactate is small molecule (0.09 kDa) and well known biomarker used in several acute clinical settings. It has important metabolic and physiological functions (153). Within the dermal compartment, lactate enters the interstitial fluid through both systemic diffusion across capillaries making detection much less technically challenging. Human detection technologies have transitioned from invasive microdialysis to minimally invasive, wearable microneedle biosensors that provide continuous, real-time measurements (19, 20, 64–66). Clinical studies validate that these sensors accurately track venous lactate concentrations with high patient tolerance and a minimal lag time of approximately five to ten minutes (66). Elevated lactate levels in cardiovascular patients serve as powerful independent prognostic indicators for serious clinical outcomes, including in heart failure and mortality following myocardial infarction (154–157). The relative ease of lactate detection in the dermal ISF is reflected in the maturity of devices described in the literature. Although no device is yet approved for medical use, lactate dermal ISF detection is arguably the closest to clinical translation.

## 7 Clinical applications and potential future impact

The development of wearable continuous sensor technology coincides with a significant advance in the analysis and handling of large data sets through machine learning. These technologies are converging with the potential to produce a paradigm shift in clinical diagnostic approaches, with a change from spot testing to continuous monitoring of known analytes. There is growing evidence that traditionally well understood clinical tests, can provide further novel clinical insights when analysed in large data sets through machine learning algorithms (158–161). This approach may help understand key areas of uncertainty in cardiovascular disease.

### 7.1 Coronary care

Although much progress has been made in the diagnosis of coronary disease using troponin and imaging tests; there remains diagnostic uncertainty in several areas that continuous monitoring may address.

In acute coronary syndromes, troponin is a key clinical test. Current diagnostic troponin algorithms perform well when patients have presented an hour after symptoms with a troponin level above or below a set concentration (8). However, many patients present outside this time frame with troponin levels between cut of values. These patients do not meet diagnostic criteria but have 4–5x higher risk of death than patients with normal troponin level (162). Current practice advocates further observation, testing and expert clinical review (8). However, this can result in delay of appropriate diagnosis and treatment requiring significant health resources. Even for patients with troponin concentrations below diagnostic cut off values, there is a 0.5% risk of 30 day MACE (162). Given a total 1.39 million patients presented with chest pain to the National Health Service (NHS) England in 2023/24 (163), these groups represent a significant diagnostic challenge to health systems.

The hypothetical application of a wearable continuous troponin sensor offers several potential advantages in these indeterminate groups: a) repeat troponin testing is performed non-invasively and continuously providing real time trend based data as a surrogate of infarction, b) *in-situ* sensor analysis removes the need for venepuncture, lab analysis and associated healthcare resources [‘lab-on-chip approach’], c) wearable sensors will enable ambulatory management of patients with sufficiently low immediate risk.

Continuous monitoring in the established acute coronary syndrome group may also offer further opportunity. Despite several landmark studies, there remains uncertainty in several areas of management for NSTEMI: invasive vs. conservative, early invasive vs. late invasive, image guided, single vs. multivessel (164–171). So far, a blanket management approach for NSTEMI patients has failed to yield convincing results, suggesting this group requires more detailed stratification that may be facilitated by data from continuous monitoring.

### 7.2 Heart failure

A key advantage of the wearable sensor is the ‘lab on chip’ concept that enables monitoring outside of hospital environments, especially for chronic conditions such as heart failure. NPs are a well-established marker of disease and potentially more informative when repeated over time (172, 173). Thus heart failure management through the use of a BNP or NT-proBNP wearable continuous sensor has many potential applications.

In stable chronic heart failure patients’ serial NP testing results in better drug optimisation and reduction of heart failure related events at least (174–176). Several studies have shown that NP directed therapy can improve outcomes (172). All of these studies have used laboratory NP testing requiring venepuncture and healthcare settings. It is hypothesised that non-invasive continuous NP monitoring will offer superior data trends, which may subsequently inform better management decisions, although robust prospective trials are required to confirm this assumption.

As discussed already, North American heart failure guidance suggests at least 2 NP tests for acute heart failure patients admitted to hospital (122). NP monitoring enhances clinician decision making in the acute setting in combination with conventional measures (fluid balance, oedema status, electrolytes). This combined approach is already effective with spot NP testing and may be further improved with continuous NP data; although there is equivocal data regarding the effect of serial NP monitoring on all-cause mortality (174–176).

Acute heart failure management in the ambulatory setting is now well established and effective (177–180). Remote systems are increasingly being used to aid more effective management with success depending on meaningful monitoring targets (181, 182). Wearable sensors can easily be linked to wireless microchips enabling mobile monitoring systems and smart phone integration. These features will enable healthcare systems to manage more patients outside of hospital as the ‘internet of things [IoT] concept takes shape globally (183).

### 7.3 Data interpretation, digital algorithms, and AI integration

The transition from episodic spot testing to high-frequency interstitial monitoring necessitates a fundamental shift in clinical data interpretation. A primary challenge in this paradigm is the significant data volume of continuous biomarker monitoring that could overwhelm traditional clinical workflows. To translate these raw data streams into actionable insights, robust digital algorithms and machine learning (ML) frameworks are essential.

There is rapidly growing evidence showing that multimodal AI integration that combines physiological data such as heart rate variability, motion sensors and electrocardiogram (ECG) signals can significantly enhance diagnostic accuracy and patient outcomes in cardiovascular care (184–192). Such systems move beyond static, population-wide thresholds by utilising ML to establish personalised baselines, identifying subtle deviations from a patient's unique physiological norm. The use of AI models to manage and contextualise large continuous biomarker datasets will therefore be key to the practical implementation of wearable sensors in the clinical setting.

A key challenge will be the potential high false-positive rates inherent to sensitive monitoring systems. In the intensive care setting, up to 99% of alarms have been identified as non-actionable, contributing to significant clinician desensitisation and alarm fatigue (193). AI-based management interventions offer a solution to this burden; recent systematic reviews indicate that intelligent algorithms can suppress false alarms by up to 92.25% and reduce total caregiver notifications by over 99% without compromising patient safety (194, 195). By filtering movement artifacts and accounting for the physiological time-lag between blood and ISF compartments, these clinical decision support tools can triage high-risk patients for urgent review while effectively monitoring lower-risk individuals in ambulatory settings.

## 8 Challenges and limitations

At present, no cardiovascular specific analyte sensor has been developed and deployed for clinical use. Although several promising technologies have been reviewed here, they will need to overcome some remaining issues for clinical acceptance. Broadly these can be split into technical, ISF-specific and clinical challenges.

### 8.1 Technical challenges

There are several ISF sampling methods available and under investigation. Each method has its own specific advantage profile. However, all methods are dependent on local factors at the area of sampling (pressure, moisture, skin/soft tissue injury) and are therefore susceptible to sampling errors. All dermal ISF methods are only able to collect microlitre quantities, of which the constituent concentrations are in the nanogram to picogram per millilitre range. Some sampling methods may also disrupt localised mechanisms and structures causing biofouling errors. Nonetheless, several sampling methods have been shown to be robust for clinical and research use, especially for small molecules.

Detection methods remain a key area for development. Of the methods reviewed here, enzymatic sensors are the most widely adopted technology in clinical use but may be limited to small molecule detection given the lack of cardiac biomarker specific enzymes. Although this may be overcome using antibody linked enzymes. Enzymatic-electrochemical detection is a proven technology and can be miniaturised to wearable sensors. Biofouling, calibration requirements and enzyme denaturation may potentially limited frequent sampling rates required for wearable sensors.

ISE detection may have some limited utility in cardiac patients but ultimately lacks specificity for more complex biomarkers. The technology is being used in elite sports monitoring with some established wearables commercially, but medical use may be limited.

Aptamer based sensors offer promising specificity advantages for the future. Further development is required to understand if the underlying challenges of this technology can be overcome. Nuclease degradation, lower affinity binding in complex fluids and immobilisation are the key issues from experiments thus far. Further human studies of complex analyte detection are required to fully evaluate aptasensors.

### 8.2 Biological challenges

Dermal ISF is a large volume fluid dispersed across the complex ECM. ISF is infused over tissues delivered by capillary blood flow and collected through venous and lymphatic capillary systems. ISF fluid is under negative pressure in normal physiological conditions. These features pose several inherent challenges: a) Sample volumes and constituents are dependent on local tissue factors and may not reflect global physiological status, b) localised ISF sample volumes are small and are not replenished, c) the complex ECM may i) trap or interact with analytes of interest making detection more difficult, ii) interfere with sensor probes causing biofouling and detection errors, d) there is an inherent time lag of blood biomarker transfer to ISF which may lead to reduce diagnostic value.

Given the array of biosensing technologies emerging, the most significant translational hurdle is likely to be whether dermal ISF is currently a sufficiently validated biological substrate for acute cardiovascular monitoring. Reliable continuous ISF collection and robust biological validation remain major bottlenecks.

Crucially, while the blood-ISF correlation is well-established and mathematically predictable for small metabolites like glucose and lactate, robust human data demonstrating consistent correlations for larger cardiovascular proteins (such as troponin and CRP) remain severely limited. The complex ECM may trap or interact with these larger analytes, exacerbating the inherent pharmacokinetic time-lag between blood and ISF. Until large-scale, prospective clinical studies definitively map these concentration gradients and prove that ISF fluctuations reliably mirror venous blood during acute cardiac events, the clinical utility of ISF as a standalone diagnostic substrate for cardiovascular care remains unproven.

### 8.3 Clinical/regulatory challenges

While this review highlights promising advances in dermal interstitial fluid (ISF) biomarker detection, there remains a significant translational gap between technological potential and demonstrated clinical benefit. To date, few studies have directly evaluated whether continuous biomarker monitoring improves diagnostic accuracy or patient outcomes in cardiovascular care.

Several studies included in this review support the potential of continuous monitoring. For example, the detection of NT-proBNP trends in saliva and urine has shown correlation with blood levels and response to heart failure treatment, suggesting that continuous monitoring could enhance disease tracking and prognostication. Similarly, troponin monitoring in ISF has demonstrated delayed but detectable trends in animal models of myocardial infarction. These findings support the feasibility of non-invasive monitoring and the potential for capturing temporal biomarker profiles beyond the capability of spot blood sampling.

However, these technologies have yet to be validated in real-world clinical environments. The shift from traditional point-in-time testing to continuous, real-time biomarker surveillance will require several paradigm changes in healthcare delivery. First, clinical teams must develop new diagnostic frameworks that incorporate biomarker trends over time rather than static thresholds. Second, robust evidence must be generated to demonstrate that real-time monitoring provides actionable data that improves clinical decision-making and patient outcomes.

Importantly, continuous monitoring may prove disruptive to existing healthcare systems. The increased volume of data generated from wearable biosensors could overwhelm clinical workflows and require substantial investment in infrastructure, data analysis tools, and clinician training. Healthcare providers may face challenges in interpreting continuous streams of biomarker data, establishing clinical significance, and avoiding false alarms.

Ultimately, demonstrating clinical value will require prospective trials to evaluate diagnostic accuracy, cost-effectiveness, and impact on outcomes. Until then, the promise of continuous cardiovascular biomarker monitoring remains compelling—but unproven.

## 9 Conclusion

The detection of cardiovascular biomarkers in dermal interstitial fluid (ISF) represents a promising frontier in the early diagnosis and management of cardiovascular disease. The minimally invasive nature and continuous sampling capabilities of dermal ISF provide distinct advantages over traditional blood-based biomarker detection, potentially improving diagnostic accuracy, broadening diagnostic windows, and enabling novel biological insights. These benefits are particularly relevant for the monitoring of biomarkers such as troponin, BNP/NT-proBNP, and emerging indicators like microRNAs, which hold significant diagnostic and prognostic value in acute coronary syndromes and heart failure.

Advances in microneedle arrays, reverse iontophoresis, and filament-based extraction methods have demonstrated the feasibility of continuous ISF sampling. Coupled with promising detection technologies—including enzyme-linked sensors, ion-sensitive electrodes, and aptamer-based sensors—there is substantial potential to develop wearable devices capable of real-time cardiovascular monitoring. The small molecule sensors (glucose, lactate, sodium, potassium) are the technically most feasible and mature technologies ready for clinical translation; although they lack specificity for cardiovascular disease detection and monitoring. More complex biomarkers such as troponin are emerging as technically detectable in the ISF but require further development in human studies. These innovations could significantly enhance patient care by facilitating early diagnosis, personalised treatment strategies, and timely interventions, ultimately improving clinical outcomes.

### 9.1 Barriers to clinical adoption

However, technical and inherent challenges remain. The low analyte concentrations in ISF, matrix effects of the extracellular environment, and the time lag in biomarker diffusion from blood to ISF present obstacles to sensor sensitivity and specificity. Additionally, biofouling, calibration stability, and localised sampling inconsistencies must be addressed to ensure reliable and reproducible measurements. Overcoming these challenges will require continued multidisciplinary research integrating materials science, bioengineering, and clinical cardiology.

### 9.2 Future direction

Future research should prioritise the validation of ISF biomarker detection in clinical populations, the refinement of sensor technologies for robust and long-term performance, and the integration of these systems with data analytics and machine learning for predictive health insights. This will require clinical trials evaluating continuous monitoring against current practice and clinical outcomes. By addressing these gaps, dermal ISF biomarker detection could revolutionise cardiovascular disease management, transitioning from reactive to proactive, data-driven patient care.

In summary, while challenges remain, the convergence of advanced sampling techniques and innovative biosensing technologies positions dermal ISF as a transformative medium for continuous cardiovascular biomarker monitoring. Realising this potential could fundamentally shift the paradigm of cardiovascular diagnostics and patient management, paving the way for more precise and timely healthcare interventions.
